# Long non-coding RNA MALAT1 exacerbates acute respiratory distress syndrome by upregulating ICAM-1 expression via microRNA-150-5p downregulation

**DOI:** 10.18632/aging.102953

**Published:** 2020-04-21

**Authors:** Meng-Ying Yao, Wei-Hong Zhang, Wen-Tao Ma, Qiu-Hong Liu, Li-Hua Xing, Gao-Feng Zhao

**Affiliations:** 1 Department of Respiratory Intensive Care Unit, The First Affiliated Hospital of Zhengzhou University, Zhengzhou 450052, P.R. China; 2 Department of Anatomy, Nursing College of Zhengzhou University, Zhengzhou 450052, P.R. China; 3 Department of Thoracic Surgery, The First Affiliated Hospital of Zhengzhou University, Zhengzhou 450052, P.R. China

**Keywords:** lung adenocarcinoma transcript 1, microRNA-150-5p, intercellular adhesion molecule-1, acute respiratory distress syndrome, pulmonary microvascular endothelial cells

## Abstract

Acute respiratory distress syndrome (ARDS) is a severe form of acute lung injury in which severe inflammatory responses induce cell apoptosis, necrosis, and fibrosis. This study investigated the role of lung adenocarcinoma transcript 1 (MALAT1) in ARDS and the underlying mechanism involved. The expression of MALAT1, microRNA-150-5p (miR-150-5p), and intercellular adhesion molecule-1 (ICAM-1) was determined in ARDS patients and lipopolysaccharide (LPS)-treated human pulmonary microvascular endothelial cells (HPMECs). Next, the interactions among MALAT1, miR-150-5p, and ICAM-1 were explored. Gain- or loss-of-function experiments in HPMECs were employed to determine cell apoptosis and inflammation. Furthermore, a mouse xenograft model of ARDS was established in order to verify the function of MALAT1 *in vivo*. MALAT1 and ICAM-1 were upregulated, while miR-150-5p was downregulated in both ARDS patients and LPS-treated HPMECs. MALAT1 upregulated ICAM-1 expression by competitively binding to miR-150-5p. MALAT1 silencing or miR-150-5p overexpression was shown to suppress HPMEC apoptosis, decrease the expressions of pro-inflammatory cytokines (IL-6, IL-1β and TNF-α) and E-selectin in HPMECs, as well as alleviated lung injury in nude mice. These findings demonstrated that MALAT1 silencing can potentially suppress HPMEC apoptosis and alleviate lung injury in ARDS *via* miR-150-5p-targeted ICAM-1, suggestive of a novel therapeutic target for ARDS.

## INTRODUCTION

Acute respiratory distress syndrome (ARDS) is a severe complication of acute lung injury that frequently occurs among intensive care unit patients, which can even lead to multiple organ dysfunction and high mortality owing to severe systemic inflammation [1, 2]. ARDS is associated with increased permeability of pulmonary microvascular endothelial cells (PMECs), increased lung weight, and loss of aerated lung tissues [3, 4]. Currently, noninvasive ventilation is one of the commonly used treatment modalities for ARDS [5]. In addition, cell therapy such as mesenchymal stem cell therapy, has been reported as a potential treatment option for this disease [6]. Developing novel therapeutics that can facilitate and enhance lung repair remains crucial to advance the management of this disease.

Emerging evidence has confirmed the relevance of lncRNAs to respiratory diseases and thus targeting lncRNAs has been highlighted as a novel therapeutic strategy for respiratory disease treatment [7, 8]. Lung adenocarcinoma transcript 1 (MALAT1) is an abundant lncRNA localized to nuclear speckles, which consists of a series of pre-mRNA processing factors [9]. The expression of MALAT1 is strongly regulated in lung adenocarcinoma and other physiological processes [10]. A high expression level of MALAT1 is associated with increased ARDS risk, disease severity, and increased mortality in patients with sepsis [11]. LncRNAs are likely to mediate the function of microRNAs (miRNAs or miRs) by regulating their gene expression, acting as endogenous sponges [12]. In particular, the overexpression of MALAT1 has been reported to sponge miR-150-5p and downregulate its expression in chondrocytes [13]. Multiple miRs have also been implicated in the progression of respiratory diseases [14]. miR-150 can exert protection against cigarette smoke-induced lung inflammation and resist airway epithelial cell apoptosis *via* downregulation of p53 [15]. Moreover, miR-150-5p is capable of suppressing the expression of proinflammatory cytokines in patients with ischemic stroke [16]. In addition, another ARDS-related factor, intercellular adhesion molecule-1 (ICAM-1) has been found to help ameliorate lung inflammation in a mouse model of ARDS after its expression is reduced [17]. ICAM-1 is a type of cell surface adhesion receptor that can promote multiple effectors/target cell interactions in tissues impacted by inflammatory or immune processes [18] and is involved in elevating the permeability of PMECs [19]. Herein, the current study was conducted to explore whether the MALAT1/miR-150-5p/ICAM-1 signaling axis is involved in mediating the biological functions of PMECs and lung injury following ARDS as well as the underlying mechanisms.

## RESULTS

### Downregulation of MALAT1 suppresses apoptosis of human pulmonary microvascular endothelial cells (HPMECs) and decreases expression of pro-inflammatory cytokines and adhesion factors in ARDS

**Figure 1:**
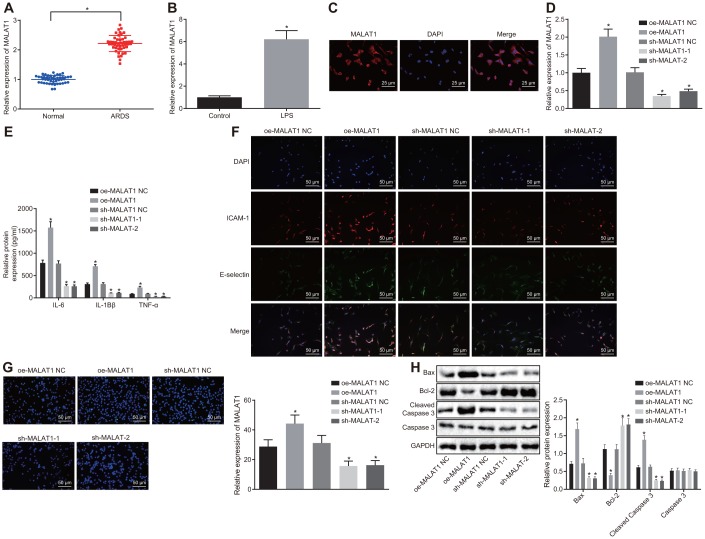
**Downregulated MALAT1 inhibits apoptosis of HPMECs while decreasing the expression of pro-inflammatory cytokines and adhesion factors.** (**A**) Expression of MALAT1 in peripheral blood samples of healthy controls (n = 46) and patients with ARDS (n = 46), as determined by RT-qPCR. * *p* < 0.05 *vs*. peripheral blood samples of healthy controls. (**B**) Expression of MALAT1 in normal and LPS-treated HPMECs, as determined by RT-qPCR. * *p* < 0.05 *vs*. the control cells. (**C**) Subcellular localization of MALAT1 in HPMECs detected by FISH (400 ×). (**D**) Expression of MALAT1 in HPMECs transfected with oe-MALAT1 or sh-MALAT1 determined by RT-qPCR. (**E**) Expression of pro-inflammatory cytokines (IL-6, IL-1β and TNF-α) in HPMECs transfected with oe-MALAT1 or sh-MALAT1 determined by ELISA. (**F**) Expression of endothelial cell adhesion molecules (E-selectin and ICAM-1) in HPMECs transfected with oe-MALAT1 or sh-MALAT1, as detected by immunofluorescence (× 200). (**G**) HPMEC apoptosis upon transfection with oe-MALAT1 or sh-MALAT1 detected by TUNEL assay (× 200). (**H**) Expression of Bcl-2, Bax, cleaved caspase 3 and Caspase3 in HPMECs transfected with oe-MALAT1 or sh-MALAT1 detected by Western blot analysis. * *p* < 0.05 *vs*. cells transfected with oe-MALAT1 NC or sh-MALAT1 NC. The data were measurement data and expressed as mean ± standard deviation. The data between two groups were compared using unpaired *t*-test and those among multiple groups were analyzed by one-way ANOVA, with Tukey's post hoc test. The cell experiment was repeated three times independently.

The expression of MALAT1 was initially determined in peripheral blood samples of 46 healthy controls and 46 patients with ARDS by reverse transcription quantitative polymerase chain reaction (RT-qPCR). Compared with healthy controls, the expression of MALAT1 was increased in patients with ARDS caused by different etiologies (*p* < 0.05) (Figure 1A). Thereafter, HPMECs were treated with lipopolysaccharide (LPS) to induce inflammation. RT-qPCR revealed that the expression of MALAT1 was increased in HPMECs upon treatment with LPS (*p* < 0.05) (Figure 1B). Fluorescence in situ hybridization (FISH) revealed that MALAT1 was mainly localized in the cytoplasm with minimal expression in the nucleus (Figure 1C). In addition, the expression of MALAT1 was found to be increased in LPS-treated HPMECs transfected with overexpression (oe)-MALAT1, and conversely, it was decreased upon MALAT1 silencing (short hairpin RNA [sh]-MALAT1-1 or sh-MALAT1-2) (*p* < 0.05) (Figure 1D).

Subsequently, the expression of proinflammatory factors (interleukin-6 [IL-6], IL-1β and tumor necrosis factor-α [TNF-α]) and endothelial cell adhesion molecules (E-selectin and ICAM-1) was detected by enzyme-linked immunosorbent assay (ELISA) (Figure 1E) and immunofluorescence (Figure 1F). Meanwhile, the apoptosis of HPMECs was analyzed by terminal deoxynucleotidyl transferase-mediated dUTP-biotin nick end labeling (TUNEL) assay (Figure 1G), and the expression of B-cell lymphoma 2 (Bcl-2), Bcl-2-associated X protein (Bax), and cleaved caspase 3 was determined by Western blot analysis (Figure 1H). The results revealed that overexpression of MALAT1 resulted in a notable increase in the expression of IL-6, IL-1β, TNF-α, E-selectin, ICAM-1, Bax and cleaved caspase 3, as well as in apoptosis rate, whereas the expression of Bcl-2 was decreased in HPMECs (*p* < 0.05). On the other hand, silencing MALAT1 led to a marked decrease in the expression of IL-6, IL-1β, TNF-α, E-selectin, ICAM-1, Bax and cleaved caspase 3 along with lowered apoptosis rate, while the expression of Bcl-2 was increased (*p* < 0.05). Taken together, the data indicated that silencing MALAT1 could inhibit apoptosis of HPMECs and reduce the expression of pro-inflammatory cytokines and adhesion factors.

### Overexpression of miR-150-5p reverses the promoting effects of MALAT1 on the progression of ARDS

**Figure 2:**
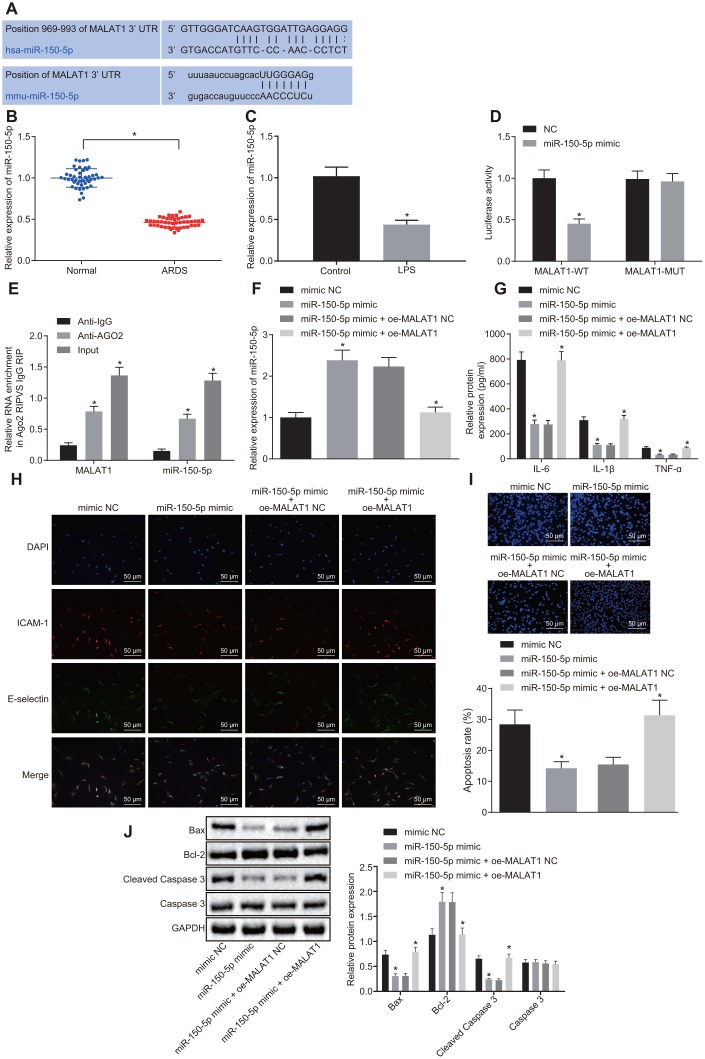
**Overexpression of miR-150-5p downregulates MALAT1 expression to suppress apoptosis of HPMECs and decrease the expression of pro-inflammatory cytokines and adhesion factors.** (**A**) The binding site between MALAT1 and miR-150-5p predicted by the starBase website; (**B**) The expression of miR-150-5p in peripheral blood samples of healthy controls (n = 46) and patients with ARDS (n = 46), as determined by RT-qPCR. * *p* < 0.05 *vs*. peripheral blood samples of healthy controls. (**C**) Expression of miR-150-5p in normal and LPS-treated HPMECs determined by RT-qPCR. * *p* < 0.05 *vs*. the control cells. (**D**) Luciferase activity of MALAT1-WT and MALAT1-MUT. * *p* < 0.05 *vs*. cells transfected with NC. (**E**) The binding of MALAT1 and miR-150-5p and the binding of MALAT1 and AGO2, as analyzed using RIP assay. * *p* < 0.05 *vs*. IgG. (**F**) Expression of miR-150-5p in HPMECs transfected with miR-150-5p mimic or miR-150-5p mimic + oe-MALAT1 determined by RT-qPCR. (**G**) Expression of pro-inflammatory cytokines (IL-6, IL-1β and TNF-α) in HPMECs transfected with miR-150-5p mimic or miR-150-5p mimic + oe-MALAT1 determined by ELISA. (**H**) Expression of endothelial cell adhesion molecules (E-selectin and ICAM-1) in HPMECs transfected with miR-150-5p mimic or miR-150-5p mimic + oe-MALAT1 detected by immunofluorescence (× 200). (**I**) HPMEC apoptosis upon transfection with miR-150-5p mimic or miR-150-5p mimic + oe-MALAT1 detected by TUNEL assay (× 200). (**J**) Expression of Bcl-2, Bax, cleaved caspase 3 and Caspase3 in HPMECs transfected with miR-150-5p mimic or miR-150-5p mimic + oe-MALAT1 detected by Western blot analysis. * *p* < 0.05 *vs*. cells transfected with miR-150-5p mimic NC or miR-150-5p mimic + oe-MALAT1 NC. The data were measurement data and expressed as mean ± standard deviation. The data between two groups were compared using unpaired *t*-test and those among multiple groups were analyzed by one-way ANOVA, with Tukey's post hoc test. The cell experiment was repeated three times independently.

The biological prediction website starBase (http://starbase.sysu.edu.cn/) predicted a binding site between miR-150-5p and MALAT1 (Figure 2A). The expression of miR-150-5p was lower in 46 patients with ARDS than that in the healthy controls (*p* < 0.05) (Figure 2B). The expression of miR-150-5p was also decreased in HPMECs following LPS treatment (*p* < 0.05) (Figure 2C). Therefore, it was speculated that MALAT1 might affect ARDS by binding to miR-150-5p. To verify this speculated relationship, dual luciferase reporter assay was conducted, and the results (Figure 2D) showed miR-150-5p mimic transfection led to a decrease in the luciferase activity of MALAT1-wild type (WT) as compared with negative control (NC) transfection, (*p* < 0.05), while the luciferase activity of MALAT1-mutant (MUT) showed no changes (*p* > 0.05). RNA binding protein immunoprecipitation (RIP) and RNA pull-down assays showed that miR-150-5p-WT could combine more MALAT1 in comparison to miR-150-5p-MUT and Bio-NC (*p* < 0.05), which further confirmed that MALAT1 could bind to miR-150-5p. Moreover, the expression of miR-150-5p was upregulated in LPS-treated HPMECs upon miR-150-5p-mimic transfection, which was negated by dual transfection with miR-150-5p mimic and oe-MALAT1 (*p* < 0.05) (Figure 2F). These results demonstrated that MALAT1 could bind with miR-150-5p and consequently reduce its expression.

ELISA and immunofluorescence (Figure 2G, 2H) revealed much lower expression of IL-6, IL-1β, TNF-α, E-selectin, and ICAM-1 in cells after treatment with miR-150-5p-mimic than treatment with miR-150-5p-mimic NC (*p* < 0.05), which was reversed by dual transfection with miR-150-5p mimic and oe-MALAT1 (*p* < 0.05). As illustrated in Figure 2I, 2J, TUNEL assay, RT-qPCR and Western blot analysis demonstrated that HPMECs treated with miR-150-5p-mimic showed a decrease in apoptosis rate and the expression of Bax and cleaved caspase 3, yet an increase in Bcl-2 expression, as compared with cells treated with miR-150-5p-mimic NC (*p* < 0.05). However, co-transfection with miR-150-5p mimic and oe-MALAT1 led to a converse trend in expression of the aforementioned factors (*p* < 0.05). Overall, the above diagrams served to illustrate that miR-150-5p overexpression was capable of rescuing the stimulated apoptosis of HPMECs and increased expression of pro-inflammatory cytokines and adhesion factors induced by MALAT1 overexpression.

### MALAT1 upregulates the expression of miR-150-5p-targeted ICAM-1 by binding to miR-150-5p

**Figure 3:**
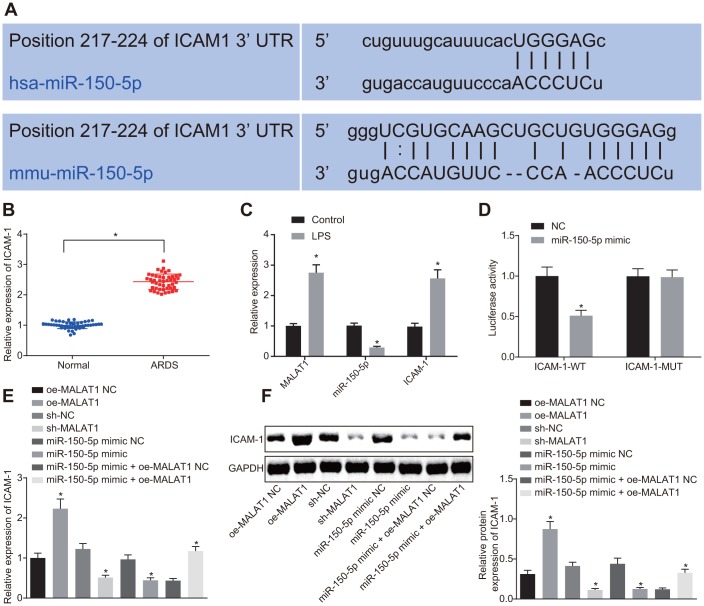
**MALAT1 competitively binds to miR-150-5p, thereby upregulating the expression of miR-150-5p-targeted ICAM-1.** (**A**) Binding sites between miR-150-5p and ICAM-1 predicted using the starBase website. (**B**) Expression of ICAM-1 in peripheral blood samples of healthy controls (n = 46) and patients with ARDS (n = 46) determined by RT-qPCR. * *p* < 0.05 *vs*. peripheral blood samples of healthy controls. (**C**) Expression of ICAM-1, MALAT1 and miR-150-5p in normal HPMECs and LPS-treated HPMECs determined by RT-qPCR. * *p* < 0.05 *vs*. the control. (**D**) The targeting relationship between miR-150-5p and MALAT1 in cells verified by dual luciferase reporter assay. * *p* < 0.05 *vs*. cells transfected with NC. (**E**) Expression of ICAM-1 in HPMECs determined by RT-qPCR. (**F**) Expression of ICAM-1 in HPMECs determined by Western blot analysis. * *p* < 0.05 *vs*. cells transfected with oe-MALAT1 NC, sh-NC, miR-150-5p mimic NC, or miR-150-5p mimic + oe-MALAT1 NC. The data were measurement data and expressed as mean ± standard deviation. The data between two groups were compared using unpaired *t*-test and those among multiple groups were analyzed by one-way ANOVA, with Tukey's post hoc test. The cell experiment was repeated three times independently.

Binding sites between miR-150-5p and ICAM-1 were predicted using the starBase website (Figure 3A). LPS-treated HPMECs exhibited high expression of MALAT1 and low expression of miR-150-5p. Moreover, ICAM-1 was highly expressed in patients with ARDS and LPS-treated HPMECs (Figure 3B, 3C). The dual luciferase reporter assay exhibited that the luciferase activity of ICAM-1-WT was notably decreased following transfection of miR-150-5p mimic (*p* < 0.05), while that of ICAM-1-MUT showed no changes (*p* > 0.05) (Figure 3D), suggesting a targeting relationship between miR-150-5p and ICAM-1. RT-qPCR and Western blot analysis showed that oe-MALAT1-transfected cells showed elevated expression of ICAM-1 in comparison to oe-MALAT1 NC-transfected cells (*p* < 0.05). In contrast, transfection with miR-150-5p mimic led to a decrease in ICAM-1 expression compared to transfection with miR-150-5p mimic NC (*p* < 0.05), which was rescued by co-transfection with miR-150-5p mimic and oe-MALAT1 (*p* < 0.05). These findings demonstrated that MALAT1 partially alleviated the inhibitory effect of miR-150-5p on ICAM-1 expression.

### Downregulation of ICAM-1 inhibits apoptosis of HPMECs and reduces the expression of pro-inflammatory cytokines and adhesion factors in ARDS

**Figure 4:**
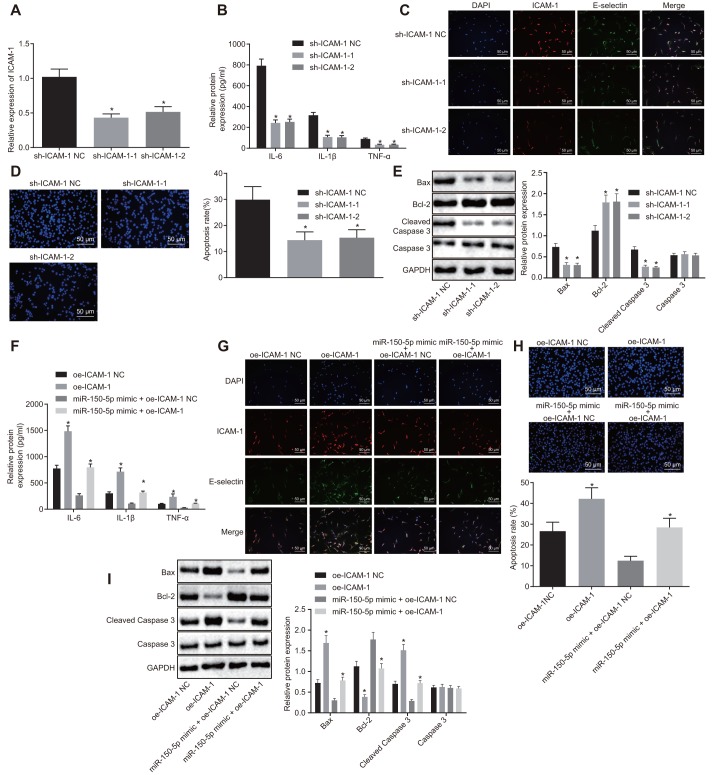
**Downregulated ICAM-1 results in inhibited HPMEC apoptosis and decreased expression of pro-inflammatory cytokines and adhesion factors.** (**A**) Expression of ICAM-1 in HPMECs. (**B**) Expression of pro-inflammatory cytokines (IL-6, IL-1β and TNF-α) in HPMECs transfected with sh-ICAM-1, as determined by ELISA. (**C**) The expression of endothelial cell adhesion molecules (E-selectin and ICAM-1) in HPMECs transfected with sh-ICAM-1 detected by immunofluorescence (× 200). (**D**) HPMEC apoptosis following transfection with sh-ICAM-1 detected by TUNEL assay (× 200). (**E**) Expression of Bcl-2, Bax, cleaved caspase 3 and Caspase3 in HPMECs transfected with sh-ICAM-1 detected by Western blot analysis. (**F**) The expression of pro-inflammatory cytokines (IL-6, IL-1β and TNF-α) in HPMECs determined by ELISA. (**G**) The expression of endothelial cell adhesion molecules (E-selectin and ICAM-1), as detected by immunofluorescence (× 200). (**H**) HPMEC apoptosis detected by TUNEL assay (× 200). (**I**) The expression of Bcl-2, Bax, cleaved caspase 3 and Caspase3 in HPMECs detected by Western blot analysis. * *p* < 0.05 *vs*. cells transfected with oe-ICAM-1 NC, sh-ICAM-1 NC or miR-150-5p mimic + oe-ICAM-1 NC. The data were measurement data and expressed as mean ± standard deviation. The data between two groups were analyzed by unpaired *t*-test and those among multiple groups were analyzed by one-way ANOVA, with Tukey's post hoc test. The cell experiment was repeated three times independently.

Next, we aimed to elucidate the possible effect of ICAM-1 on the progression of ARDS. Compared with sh-NC-transfected cells, the expression of ICAM-1 was reduced in cells following transfection with sh-ICAM-1-1 or sh-ICAM-1-2 (Figure 4A). ELISA (Figure 4B–4F), immunofluorescence (Figure 4C), TUNEL assay (Figure 4D), and Western blot analysis (Figure 4E) revealed that relative to sh-ICAM-1 NC transfection, transfection with sh-ICAM-1-1 or sh-ICAM-1-2 resulted in decreased expression of IL-6, IL-1β, TNF-α, E-selectin, ICAM-1, Bax and cleaved caspase 3, in addition to reduced apoptosis rate, whereas Bcl-2 expression was increased (*p* < 0.05).

Moreover, there was an upward trend in the expression of IL-6, IL-1β, TNF-α, E-selectin, ICAM-1, Bax and cleaved caspase 3 in addition to elevated apoptosis rate while Bcl-2 expression was reduced in cells transfected with oe-ICAM-1 (*p* < 0.05). Relative to treatment with miR-150-5p mimic + oe-ICAM-1-NC, treatment with miR-150-5p mimic + oe-ICAM-1 resulted in a significant elevation in the expression of IL-6, IL-1β, TNF-α, E-selectin, ICAM-1, Bax and cleaved caspase 3, along with an increase in apoptosis rate, yet decreased Bcl-2 expression (*p* < 0.05) (Figure 4G–4I). These results demonstrated that the depletion of ICAM-1 restrained apoptosis of HPMECs and reduced the expression of pro-inflammatory cytokines and adhesion factors.

### Downregulation of MALAT1 or overexpression of miR-150-5p alleviates lung injury

**Figure 5:**
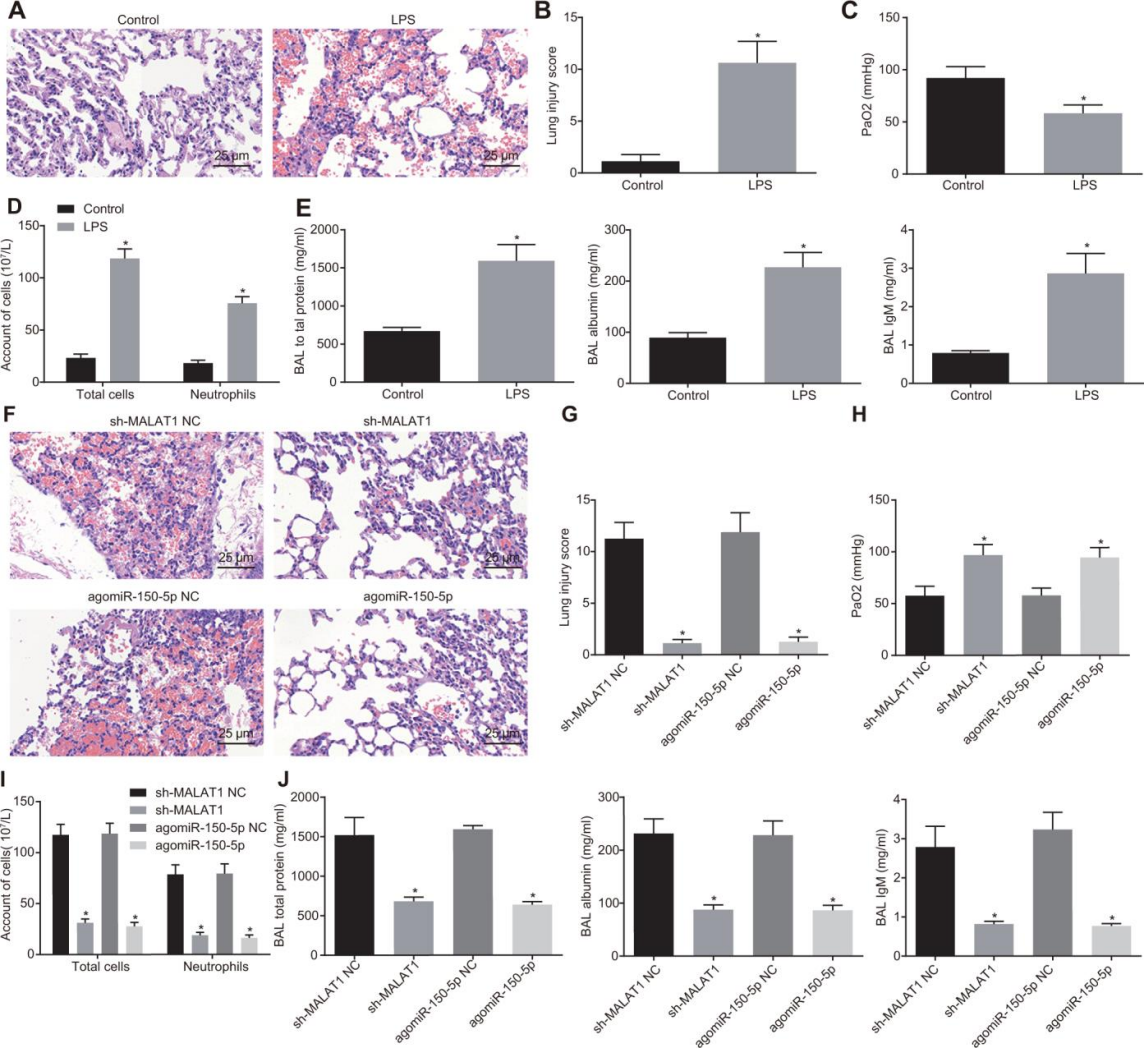
**Downregulated MALAT1 or upregulated miR-150-5p alleviates lung injury.** (**A**) HE staining (× 400) of lung tissues from healthy control and ARDS mice. * *p* < 0.05 *vs*. healthy control mice. (**B**) Lung tissue injury score of healthy control and ARDS mice. * *p* < 0.05 *vs*. healthy control mice. (**C**) PaO2 of healthy control and ARDS mice. * *p* < 0.05 *vs*. healthy control mice. (**D**) The number of total cells, neutrophils, and macrophages in BAL of healthy control and ARDS mice. * *p* < 0.05 *vs*. healthy control mice. (**E**) The concentration of total protein, albumin, and IgM in BAL of healthy control and ARDS mice. (**F**) HE staining analysis of lung tissues of ARDS mice after injection with different lentiviruses (× 400). (**G**) Lung tissue injury score of ARDS mice after injection with different lentiviruses. (**H**) PaO2 in arterial blood of ARDS mice after injection with different lentiviruses. (**I**) The number of total cells, neutrophils and macrophages in BAL of ARDS mice. (**J**) The concentration of total protein, albumin, and IgM in BAL of ARDS mice after injection with different lentiviruses. * *p* < 0.05 *vs*. cells transfected with sh-MALAT1 NC or agomiR-150-5p NC. The data were measurement data and expressed as mean ± standard deviation. The data between two groups were analyzed by unpaired *t*-test. n = 8 for mice following each treatment.

Finally, we aimed to explore the role of MALAT1 and miR-150-5p in ARDS *in vivo*. Hematoxylin-eosin (HE) staining analysis showed that mouse lung tissues underwent a series of typical pathological changes after treatment with LPS (Figure 5A, 5B). Next, the partial pressure of oxygen (PaO2) (Figure 5C–5E) in arterial blood, bronchoalveolar lavage (BAL) cell count, and neutrophil cell count, as well as the concentration of total protein, albumin and immunoglobulin M (IgM) in mice (Figure 5D–5G) were measured. In addition, LPS-treated mice presented with increases in pathological score, total inflammatory cells and neutrophils in BAL, and concentration of total protein, albumin, and IgM in BAL while PaO2 was decreased (*p* < 0.05). These findings indicated that LPS induced pneumonia and lung injury in mice.

Subsequently, the mice were injected with different lentiviruses to alter the expression of miR-150-5p and MALAT1. As shown in Figure 5F–5J, as compared with sh-NC or agomiR-150-5p NC treatment, mice treated with sh-MALAT1 or agomiR-150-5p showed reductions in lung injury, pathological score, total inflammatory cells and neutrophils in BAL, total protein, albumin and IgM in BAL, whereas PaO2 was elevated (*p* < 0.05). These results demonstrated that the downregulation of MALAT1 or overexpression of miR-150-5p could alleviate LPS-induced pulmonary inflammation and enhance lung permeability.

## DISCUSSION

ARDS is a multifactorial disorder and a severe manifestation of acute lung injury characterized by high morbidity and mortality rates [20]. Studies have documented the prognostic potential of lncRNAs in ARDS [21, 22]. The present study aimed to explore the effects of MALAT1 on the progression of ARDS. Our results demonstrated that downregulation of MALAT1 could potentially decrease the expression of ICAM-1 by up-regulating miR-150-5p, thereby suppressing the apoptosis of HPMECs and alleviating lung injury in ARDS mice.

Our initial findings demonstrated upregulated MALAT1 and ICAM-1 alongside downregulated miR-150-5p in peripheral blood samples of patients with ARDS and LPS-treated HPMECs. LncRNAs have been found to be upregulated in diverse intricate human diseases such as pulmonary fibrosis and lethal lung developmental disorders [23, 24]. Similar to our findings, MALAT1 has been shown to be significantly increased in the peripheral blood mononuclear cells with infection exposure and treated with LPS in neonatal respiratory distress syndrome [25]. In addition, a recent study has revealed that MALAT1 is highly expressed in the plasma of ARDS patients and peripheral blood mononuclear cells [26]. Patients with ARDS show significantly reduced serum miR-150 expression, which has been found negatively associated with disease severity and 28-day survival [27]. Furthermore, the inhibition of ICAM-1 has been found to help ameliorate LPS-induced ARDS in rats [28], which suggests an increased ICAM-1 level in ARDS.

We also found that MALAT1 could downregulate miR-150-5p by competitively binding to it, thereby upregulating the expression of ICAM-1, a target of miR-150-5p. LncRNAs have been widely reported to serve as miR sponges, thereby regulating the expression of their target genes of miRs by serving as competing endogenous RNAs (ceRNAs) [29]. For instance, MALAT1 is able to sponge miR-124 and thus facilitates cell apoptosis in Parkinson’s disease [30]. In addition, MALAT1 has been found to promote cell apoptosis in testicular ischemia-reperfusion injury through upregulation of TRPV4 expression by sponging miR-214 [31]. By functioning as a sponge or as a competing endogenous noncoding RNA for miR-150-5p, lncRNA FOXD3-AS1 promotes hyperoxia-induced lung epithelial cell death [32]. In accordance with the findings from the current study, miR-150 has been demonstrated to be a target of MALAT1 in airway smooth muscle cells and MALAT1 can augment the expression of eIF4E, a target of miR-150, as a ceRNA for miR-150 [33].

Another key observation of the current study indicated that downregulation of MALAT1 or overexpression of miR-150-5p inhibited the apoptosis of HPMECs, suppressed the inflammatory response (reflected by decreased expression of Bax, cleaved caspase 3, IL-6, IL-1β, TNF-α and increased expression of Bcl-2) and decreased the expression of adhesion factors ICAM-1 and E-selectin *in vitro*, and also alleviated lung injury in ARDS *in vivo*. These observations are supported by existing evidence. The downregulation of MALAT1 has earlier been shown to inhibit the apoptosis of hypoxic/reoxygenated human umbilical vein endothelial cells *in vitro* [34], which is consistent with our findings. TNF-induced miRs can mediate TNF-induced expression of both E-selectin and ICAM-1 in human endothelial cells and thus provide a feedback control of inflammation [35]. The upregulation of exogenous miR-150 is capable of contributing to an enhancement in HMEC-1 cell migration in atherosclerosis [36], which is aligned with our findings. Overexpression of MALAT1 promotes cell apoptosis and then deteriorates lung injury through sponging of miR-425 during ARDS [26]. In LPS-induced acute kidney injury, silencing MALAT1 elevates the expression of miR-146a, leading to repression of the pro-inflammatory nuclear factor-κB (NF-κB) pathway and its downstream transcription factors [37]. Increased miR-150 decreases neutrophil counts and production of inflammatory cytokines IL-1β, IL-6, and TNF-α along with levels of total protein, albumin and IgM in the BAL fluid in LPS-induced acute lung injury mice *in vivo*, as well as alleviating LPS-induced A549 cell apoptosis *in vitro* [27]. Furthermore, blocking pulmonary ICAM-1 expression potentially alleviates lung injury in diet-induced pancreatitis [38]. ICAM-1 has also been reported to induce IL-6 and TNF-α production in a Kupffer cell-dependent manner during liver regeneration following hepatectomy [39]. In addition, retreatment with anti-ICAM-1 antibody can reduce the extent of acinar cell damage and inhibit their apoptosis [40]. On the basis of the aforementioned information, we reasoned that MALAT1 could upregulate the expression of ICAM-1 by binding to miR-150-5p, thereby suppressing the apoptosis of HPMECs and alleviating lung injury in ARDS.

**Figure 6:**
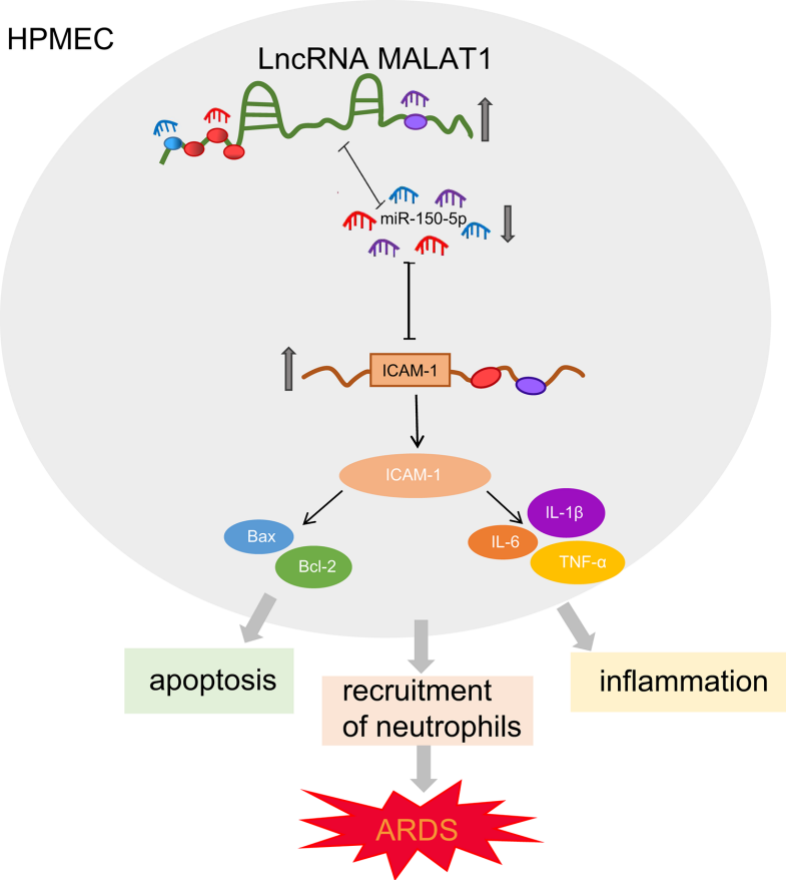
**Schematic diagram illustrating MALAT1 upregulates ICAM-1 expression by binding to miR-150-5p and consequently stimulates HPMEC apoptosis and neutrophil adhesion, ultimately accelerating pulmonary inflammation and reducing lung permeability in ARDS mice.**

To conclude, the present study demonstrated that MALAT1 silencing could suppress HPMEC apoptosis and alleviate lung injury in ARDS *via* the miR-150-5p-mediated ICAM-1 axis (Figure 6), indicating that targeting MALAT1 may serve as a promising therapeutic strategy for ARDS. Our work thus identifies the evolutionarily MALAT1/miR-150-5p/ICAM-1 signaling as a major molecular pathway in the control of ARDS. Further investigations into the interaction between MALAT1, miR-150-5p, and ICAM-1 are still required to fully elucidate the specific mechanisms of MALAT1 in ARDS.

## MATERIALS AND METHODS

### Clinical blood collection

We enrolled forty-six patients with ARDS who were hospitalized at the respiratory department, emergency department, department of critical care medicine or general surgery department of The First Affiliated Hospital of Zhengzhou University from January 2017 to June 2018 in this study. Based on their PaO2/FiO_2_ ratios on the day when they were diagnosed with ARDS, the patients were classified into 3 groups: mild (200 < PaO2/FiO_2_ ≤ 300 mm Hg; n = 15), moderate (100 < PaO2/FiO_2_ ≤ 200 mm Hg; n = 22) and severe (PaO2/FiO_2_ < 100 mm Hg; n = 9) groups. Meanwhile, 46 age-matched healthy individuals were recruited as healthy controls. Based on the data obtained from medical history and physical examination, none of the healthy controls had any symptoms or signs of pulmonary discomfort, history of underlying diseases, or pulmonary abnormalities in routine chest X-ray examination. Peripheral venous blood (2 mL) was collected from all participants, which was then subjected to ethylenediaminetetraacetic acid (EDTA) anticoagulation and centrifuged at 3000 rpm for 5 minutes. The upper-layer serum was stored at -80°C.

### Cell treatment

HPMECs were purchased from ScienCell Research Laboratories (San Diego, CA, USA), and cultured in endothelial culture medium (ECM) at 37°C in 5% CO_2_. Cells were treated with LPS (1 mg/L) for 24 hours and then collected. Then pCMV-Flag plasmids containing oe-MALAT1, sh-MALAT1, sh-NC, miR-150-5p mimic, miR-150-5p mimic NC, oe-ICAM-1, oe-NC or sh-ICAM-1 were constructed by Shanghai Sangon Biotechnology Co., Ltd. (Shanghai, China). HPMECs were subsequently inoculated into a 24-well plate and transfected with these plasmids in accordance with the instructions of the Lipofectamine 2000 kit (Invitrogen, Carlsbad, California, USA).

### Fish

The subcellular localization of MALAT1 and miR-150-5p in HPMECs was detected using a FISH kit [RiboTM lncRNA FISH Probe Mix (Red)] in accordance with the kit instructions. In short, cells were inoculated into a 24-well culture plate at a density of 6 × 10^4^ cells/well. When cell confluence reached about 80%, the cells were rinsed with phosphate buffer saline (PBS) and fixed with 4% paraformaldehyde at room temperature. After being treated with protease K (2 μg/mL), glycine, and ethylphthalide reagent, the cells were incubated with 250 μL pre-hybridization solution at 42°C for 1 hour. Next, the pre-hybridization solution was removed, and the cells were incubated with 250 μL hybridization solution containing probes (300 ng/mL) at 42°C overnight. After three washes with Tris-buffered saline Tween-20 (TBST), the cells were stained with 4',6-diamidino-2-phenylindole (DAPI) dyeing solution (1 : 800) diluted by PBS-Tween-20 (PBST) for 5 minutes. Finally, the cell slides were blocked using anti-fade mounting medium and 5 randomly selected visual fields were observed and photographed under a fluorescence microscope (Olympus, Tokyo, Japan) [41].

### Dual luciferase reporter assay

The starBase website (http://starbase.sysu.edu.cn/) screened miR-150-5p as the candidate miRNA of MALAT1, and ICAM-1 as a potential target gene of miR-150-5p. Then dual luciferase reporter assay was used to verify the predicted results. In brief, HPMECs were cultured in a 24-well plate and the luciferase reporter plasmids WT MALAT1 plasmid (MALAT1-WT-Luc), MUT MALAT1 plasmid (MALAT1-MUT-Luc), WT ICAM-1 plasmid (ICAM-1-WT-Luc) and MUT ICAM-1 plasmid (ICAM-1-MUT-Luc) were constructed. Next, the MALAT1-WT-Luc, MALAT1-Mut-Luc, ICAM-1-WT-Luc, or ICAM-1-MUT-Luc plasmids were co-transfected into HPMECs with miR-150-5p mimic or miR-150-5p mimic NC, respectively, using LipofectamineTM 2000 (Invitrogen, Carlsbad, California, USA) following the manufacturer’s instructions. After 24 h of culture, the cells were collected. Thereafter, the luciferase activity was detected at 560 nm utilizing a Dual Luciferase Reporter Assay Kit (Promega, Madison, WI, USA) and a microplate reader (MK3, Thermo Fisher Scientific, Rockford, IL, USA).

### RT-qPCR

Total RNA was extracted from the cultured cells using a TRIzol reagent (Invitrogen, Carlsbad, CA, USA), and the purity and concentration of the extracted RNA were measured using a Nano-Drop ND-1000 spectrophotometer. The obtained RNA was then reverse transcribed into complementary DNA (cDNA) according to the instructions of the PrimeScript RT reagent kit (Takara, Otsu, Shiga, Japan). Next, RT-qPCR was performed with a SYBR®Premix Ex TaqTM II kit (Takara, Otsu, Shiga, Japan) on an ABI 7500 instrument (Applied Biosystems, Foster City, CA, USA). The primer sequences (Table 1) for MALAT1, miR-150-5p, ICAM-1 and glyceraldehyde-3-phosphate dehydrogenase (GAPDH) were designed and synthesized by Shanghai Sangon Biotechnology Co., Ltd. (Shanghai, China). The relative expression of the target gene was calculated using the 2^-ΔΔCt^ method with GAPDH serving as the internal reference [42–45].

**Table 1:** Primer sequences for RT-qPCR.

**Gene**	**Forward sequence (5'-3')**	**Reverse sequence (5'-3')**
MALAT1	AAAGCAAGGTCTCCCCACAAG	GGTCTGTGCTAGATCAAAAGGC
miR-150-5p	GTCTCCCAACCCTTGTAC	TATCCAGTGCGTGTCGTG
ICAM-1	GCCCGAGCTCAAGTGTCTAA	GGAGAGCACATTCACGGCA
GADPH	GGTGGTCTCCTCTGACTTCAACA	GTGGTCGTTGAGGGCAATG

Notes: RT-qPCR, reverse transcription quantitative polymerase chain reaction; MALAT1, lung adenocarcinoma transcript 1; miR-150-5p, microRNA-150-5p; ICAM-1, intercellular adhesion molecule-1; GADPH, gyceraldehyde-3-phosphate dehydrogenase.

### RIP assay

The binding of MALAT1 and Argonaute2 (AGO2) protein was detected using a RIP kit (Millipore Corp of Billerica, Massachusetts, USA). Briefly, HPMECs were lysed with radioimmunoprecipitation assay (RIPA) lysis buffer (P0013B, Beyotime Biotechnology Co., Shanghai, China) on ice bath for 5 minutes, followed by centrifugation at 14000 rpm for 10 minutes to collect the supernatant. One portion of the cell extract was used as input, and the other portion was co-precipitated with antibody. Next, 50 μL magnetic beads were collected from each co-precipitation reaction system, washed, and re-suspended in 100 μL RIP wash buffer. Next, 5 μL antibody was added to the magnetic beads and incubated for 30 minutes at room temperature for binding. The magnetic beads-antibody complex was washed, re-suspended in 900 μL RIP wash buffer, and then incubated overnight at 4°C with 100 μL cell extract. The samples were subsequently placed on magnetic pedestals to collect bead-protein complexes. The precipitated complex and Input were treated with protease K, followed by extraction of RNA for subsequent RT-qPCR detection. The antibodies used in RIP included rabbit anti-human AGO2 (ab186733; dilution ratio of 1 : 50, Abcam Inc., Cambridge, MA, USA) and rabbit anti-human immunoglobulin G (IgG; ab109489; dilution ratio of 1 : 100, Abcam Inc., Cambridge, MA, USA) which served as NC.

### RNA-pull down assay

Biotinylated WT-bio-miR-132 and MUT-bio-miR-132 (50 nM each) were each transfected into HPMECs. After 48 hours, the cells were collected and incubated with specific lysis buffer (Ambion, Austin, Texas, USA) for 10 minutes. Next, the lysate was co-incubated with M-280 streptavidin magnetic beads (S3762, Sigma-Aldrich Chemical Company, St Louis MO, USA) that had been pre-coated with RNase-free bovine serum albumin (BSA) and yeast transfer RNA (tRNA; RNABAK-RO, Sigma-Aldrich Chemical Company, St Louis MO, USA) at 4°C for 3 hours. Finally, the enrichment of MALAT1 was detected using RT-qPCR.

### Western blot analysis

Total protein was extracted from clinical tissues or cells by lysis using RIPA lysis buffer (P0013B, Beyotime Biotechnology Co., Shanghai, China) containing phenylmethylsulfonyl fluoride (PMSF) and phosphatase inhibitor. The concentration of the extracted protein was determined using a bicinchoninic acid (BCA) kit (Beyotime Biotechnology Co., Shanghai, China). A total of 30 ug proteins were subjected to separation using sodium dodecyl sulfate-polyacrylamide gel electrophoresis, and then transferred onto a nitrocellulose membrane using the wet transfer method. Next, the membrane was blocked with 5% skimmed milk prepared with TBST for 1.5 hours, and then incubated with the following primary antibodies: rabbit anti-human ICAM-1 (ab109361; dilution ratio of 1 : 2000, Abcam, UK), rabbit anti-human B-cell CLL/Lymphoma 2 (Bcl-2; ab32124; dilution ratio of 1 : 1000, Abcam, UK), rabbit anti-human Bcl-2-associated X protein (Bax; ab32503; dilution ratio of 1 : 5000, Abcam, UK), rabbit anti-human Caspase3 (ab13847, dilution ratio of 1 : 500, Abcam, UK), cleaved caspase 3 (Asp175, dilution ratio of 1 : 1000, Cell Signaling, Beverly, USA) [46] and rabbit anti-human GAPDH (ab9485, dilution ratio of 1 : 2500, Abcam, UK) at 4°C overnight. The following day, after three TBST rinses (15 min/time), the membrane was re-probed with horseradish peroxidase-labeled secondary goat anti-rabbit IgG (ab205718, dilution ratio of 1 : 2000 - 1 : 50000) at room temperature for 2 hours. Following three TBST rinses (15 min/time), the membrane was developed with an enhanced chemiluminescence reagent and photographed using the SmartView Pro 2000 (UVCI-2100, Major Science, Saratoga, CA, USA). The band intensities were then analyzed utilizing the Quantity One software [47].

### ELISA

The levels of IL-6, IL-1β, and TNF-α were determined in cell lysate in each group using ELISA kits (Rapidbio, Inc., West Hills, CA, USA) according to the kit instructions. In brief, the antigen was diluted with coating diluent at a dilution ratio of 1 : 20. Next, 100 μL standard dilution solution was added to each well, followed by overnight incubation at 4°C. The diluted samples were added into the reaction wells of the ELISA plate (100 μL in each well). Negative and positive controls were set. Each well was added with 100 μL enzyme conjugates that had been diluted with sample dilution solution, followed by 30-minute reaction at 37°C. Next, 100 μL horseradish peroxidase substrate solution was added, followed by coloration at 37°C for 10 to 20 minutes. In the event of significant color changes in the positive control or slight color changes in the NC, 50 μL termination solution was added to each well to terminate the reaction. The optical density (OD) of each well was then measured at a wavelength of 450 nm using the Spectramax M5 microplate reader (Molecular Devices, Sunnyvale, CA, USA).

### TUNEL assay

TUNEL staining was performed using an in situ apoptosis detection kit (Chemicon International Inc., Temecula, CA, USA). In brief, the transfected HPMECs were pretreated with 10 nmol/L docetaxel (DTX) for 24 hours. The cells were rinsed and stained according to the kit instructions. A fluorescence microscope (Axiovert 200, Carl Zeiss, Oberkochen, Germany) was used to observe and photograph the cells. The number of positive-stained cells was counted under an EVOS FL microscope (Thermo Fisher Science, Waltham, MA, USA).

### Immunofluorescence

Cells were fixed with 4% paraformaldehyde and then incubated with monoclonal antibodies against ICAM-1 and E-selectin (Abcam Inc., Cambridge, UK), respectively. The cells were then incubated with fluorescent dye-labeled secondary antibody (AF555; Abcam Inc., Cambridge, MA, USA), followed by DAPI staining. The fluorescence intensities of AF555 and AF647 were recorded at an excitation wavelength of 552 nm and 638 nm and an emission wavelength of 570 nm and 665 nm, respectively. At last, the protein levels of E-selectin and ICAM-I in cells were determined.

### Establishment of acute lung injury mouse models

A total of 40 C57BL/6 WT mice purchased from Vital River (Beijing, China) were anesthetized and underwent oral intubation. Next, mouse models of acute lung injury were established by intratracheal instillation of LPS (1.5 mg/kg). The healthy control mice were injected with NaCl solution *via* the tail vein. The lentiviruses of agomiR-150-5p-NC, agomiR-150-5p (20 mg/kg; Sigma-Aldrich Chemical Company, St Louis MO, USA) and sh-MALAT1 NC, sh-MALAT1 (10 mg/kg; GenePharma, Shanghai, China) were intratracheally injected into the mice (n = 8 for each treatment). One day after injection, the mice were stimulated by LPS. On the 21^st^ day after LPS stimulation, the mice were euthanized and their lung tissues were collected.

### HE staining and arterial blood gas analysis

Paraffin-embedded lung tissues were sliced into 5-μm-thick sections, dewaxed with xylene, and hydrated with gradient ethanol. The sections were stained with hematoxylin for 7 minutes, treated with 95% ethanol for 5 seconds, hydrated with gradient ethanol, and stained with eosin for 1 minute. The sections were then cleared with xylene, dried, and finally mounted with neutral balsam. The histological changes were observed under an optical microscope. Lung injury scores were assessed based on the following criteria [48]: (a) alveolar congestion; (b) erythrocyte exudation; (c) neutrophil exudation or aggregation in alveoli; (d) alveolar wall thickening and hyaline membrane formation. Based on severity, the disease was scored as the following four grades: grade 0, non-invasive or very mild; grade 1, mild; grade 2, moderate; grade 3, severe; grade 4, very serious. The sum of the scores for each item was considered as the total of lung injury score. Arterial blood samples were collected and the PaO2 was measured using an automated blood gas analyzer (GEMPREMIRE3000, MA, USA).

### Cell counting and measurement of albumin and IgM

BAL was conducted by injecting 0.9% sodium chloride containing 0.5 mL of EDTA (0.6 mg/L) into the lung of the mice. Bronchoalveolar lavage fluid (BALF) was retrieved *via* aspiration and then placed on ice. The total number of BALF cells was counted using a blood cell analyzer. The neutrophil count was calculated by multiplying the percentage of neutrophils in BALF and the number of total cells. BALF was retrieved and centrifuged at 800 g for 6 minutes. The levels of albumin and IgM were determined using a mouse specific albumin ELISA kit (ALPCO Diagnostic, Salem, NH, USA) and a mouse specific IgM ELISA kit purchased from Bethyl Laboratory (Montgomery, TX, USA), respectively.

### Statistical analysis

Statistical analyses were conducted using the SPSS 21.0 statistical software (IBM Corp. Armonk, NY, USA). Measurement data obeying normal distribution were summarized as mean ± standard deviation. Comparisons between two groups were conducted using unpaired *t*-test and those among multiple groups were conducted by one-way analysis of variance (ANOVA), followed by Tukey’s post hoc tests with corrections for multiple comparisons. A value of *p* < 0.05 indicated the difference was statistically significant.

### Ethics statement

The current study was conducted after approval by the ethics committee of The First Affiliated Hospital of Zhengzhou University (NO. 201611042). Signed written informed consent was obtained from all participants or their guardians prior to sample collection. Animal experiments were conducted according to the international convention on laboratory animal ethics and relevant national regulations (NO. 201812009), and all efforts were made to minimize the suffering of the included animals.
